# Individual phenotypic variances in a family with Avellino corneal dystrophy

**DOI:** 10.1186/1471-2415-13-30

**Published:** 2013-07-09

**Authors:** Zihret Abazi, Lidija Magarasevic, Ivana Grubisa, Dusica Risovic

**Affiliations:** 1 Eye Clinic, Zvezdara University Medical Center, 161 Dimitrija Tucovica Street, 11000, Belgrade, Serbia; 2 Department of Ophthalmology, Health Center “Palilula”, Belgrade, Serbia; 3 Department of Human Genetics and Prenatal Diagnostics, Zvezdara University Medical Center, Belgrade, Serbia; 4 Faculty of Medicine, University of Belgrade, Belgrade, Serbia

**Keywords:** Cornea, Avellino corneal dystrophy, R124H mutation, AS-OCT

## Abstract

**Background:**

Avellino corneal dystrophy (ACD) is an autosomal dominant disorder, characterized by the presence of deposits in the anterior stroma, and results from a specific mutation (R124H) in the transforming growth factor beta-induced gene (TGFBI). This report presents corneal dystrophy of the Bowman layer as a rare phenotypic appearance of ACD and a high intra-familial variability of phenotype in patients with ACD.

**Case presentation:**

A 56 year-old Caucasian woman with recurrent corneal erosions was diagnosed with corneal dystrophy of the Bowman layer after a clinical examination. Optical coherence tomography of the anterior segment (AS-OCT) mainly demonstrated deposits in the Bowman layer and a few deposits in the superficial stroma. Her son, a 36 year-old man, has a typical clinical presentation of ACD with all the deposits arranged in stromal layers. In his case, the opacities resemble snowflakes between the granular deposits, and AS-OCT shows large, snowflake-like deposits in the superficial and deep stroma without accumulation in the Bowman layer. Genetic screening in both cases shows the heterozygous R124H mutation in the TGFBI gene.

**Conclusion:**

The clinical finding of the granular-lattice corneal dystrophy in which deposits are located in the Bowman layer may be an atypical presentation of ACD. This paper demonstrates a high degree of variability in the quantity and form of deposits between ACD heterozygotes. This is the first description of Avellino corneal dystrophy in the Balkans and in Serbia.

## Background

Granular corneal dystrophy type II (CGD2, OMIM 607541), also called Avellino corneal dystrophy (ACD) or combined granular-lattice corneal dystrophy, is a rare form of autosomal dominant corneal dystrophy. The corneal opacities are result of a specific mutation (R124H) in transforming growth factor beta-induced gene (TGFBI, OMIM 601692, formerly called BIGH3) [1]. Mutations in the same gene also cause Thiel-Behnke (602082), Reis-Bücklers (608470), granular (Groenouw) type I (121900), lattice type I (122200) and epithelial basement membrane dystrophy (121820) [2].

The combined features of lattice and granular dystrophies in the same cornea resulting from mutations in the same gene raises the questions of validity of relying solely on clinical and histological evidence to classify disease. Modern genotyping now enables greater accuracy in the nosology and the International Committee for Classification of Corneal Dystrophies (IC3D) has already incorporated this information into their recent reclassification of these dystrophies [3]. Initial clinical symptoms of the heterozygous form of ACD appear during the first or second decade of life. ACD is characterized clinically by the corneal opacities that are shaped like snowflakes, discs, stars, and rings. Corneal opcitities are a bilateral formation of discrete, asymmetric, focal, grey-white granular and lattice deposits in the anterior stroma of cornea with clear areas between these deposits. There is a considerable variation of the nature and quantity of the stromal deposits both within and among families, a common characteristic of autosomal dominant disorders. In this case report, profound differences exist in the severity of the phenotypic expression within the same family, as well an atypical localization of deposits, which is in the epithelium and the Bowman layer.

### Ethical approval

Written informed consent was obtained before initiation of the exam. The cases study was approved by the ethics committee Zvezdara University Medical Center according to the principles of good clinical practice and with the ethical principles of the Declaration of Helsinki.

## Case presentation

### Case 1

A 56-year-old woman had a history of progressively decreasing visual acuity, recurrent corneal erosions and mild foreign body sensation in both eyes for about a year. Her medical history included rheumatoid arthritis and high blood pressure (hypertension). She was on therapy: methotrexate, folic acid, ibuprofen and ramipril. The patient complained of decreased vision, photosensitivity, and pain in both eyes.

**Figure 1:**
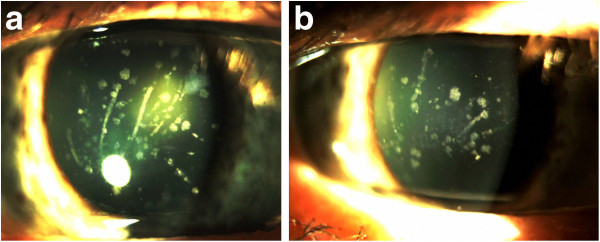
Slit-lamp photographs from Case 1 (Panel a: right eye; Panel b: left eye) shows multiple gray-white and bilateral breadcrumb-like and lattice-like opacities of the Bowman layer and anterior stroma.

**Figure 2:**
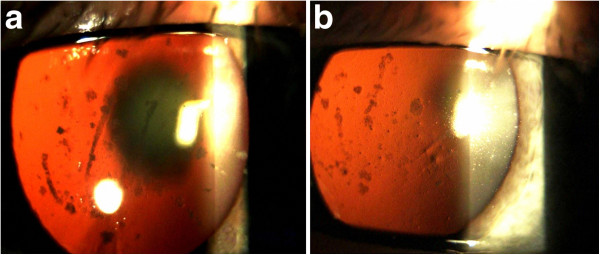
Slit-lamp photographs in retroillumination (Panel a: right eye; Panel b: left eye) shows corneal deposits and central nuclear cataract which is more pronounced in the right eye.

Slit lamp examination revealed corneal erosions and large number of gray-white central granular opacities with subepithelial haziness involving the Bowman layer and the superficial stroma (Figure 1a). Bowman’s layer appeared to be glossy and thickened with dense gray-white granular and lattice opacities in a string of breadcrumb-like arrangement (Figure 1b). The endothelium and Descemet’s membrane appeared normal. An irregularity of the epithelium was stained with fluorescein. Anterior segment examination revealed a central nuclear cataract and normal iris appearance in both eyes (Figure 2a, 2b). The intraocular pressures (IOP) by Goldmann applanation tonometer (GAT) were 16 mm Hg in both eyes. Dilated fundus examination revealed a normal, healthy fundus in both eyes. The patient’s best corrected visual acuity was 6/60 in the right eye and 6/18 in the left eye. The above findings suggested corneal dystrophy of the Bowman layer in both eyes with recurrent corneal erosions. The treatment plan included one drop of lubricant drops for both eyes, four times per day.

**Figure 3:**
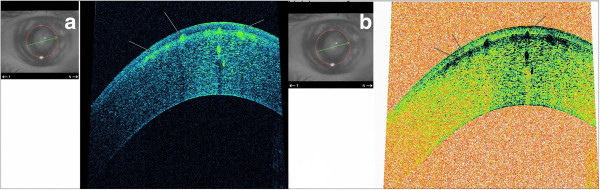
**AS-OCT asterisk scan (Panel a: classic tomogram) revealed accumulation of deposits both in the Bowman layer and superficial stroma, on cross section they have a string of pearls-like shape.** The inverse modification of tomogram with AS-OCT **(Panel b)** clear shows highly reflective deposits which are located in the epithelium, involving Bowman’s layer and anterior stroma.

**Figure 4:**
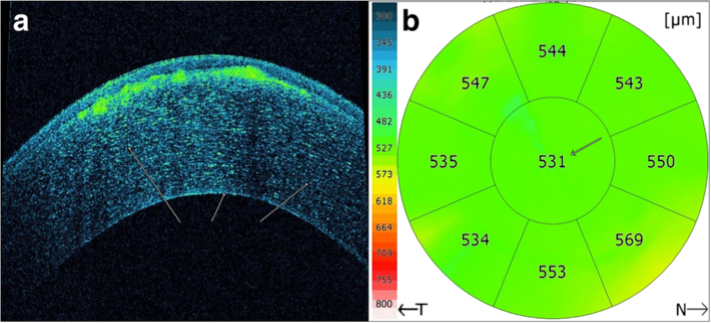
**AS-OCT scan (Panel a) shows normal deeper layers of the stroma, Descemet’s membrane and the endothelium and no deposits in these layers.** The **(Panel b)** corneal pachymetry mapping with AS-OCT showed normal value of the CCT (531 μm).

After medical treatment of corneal erosions, AS-OCT was performed (SOCT Copernicus HR®). The cross-sectional AS-OCT images (scanning programs: asterisk; axial resolution: 5 μm; scan width: 4 mm; scan depth: 2 mm) with the best quality were further analyzed using software provided by the manufacturer. AS-OCT finding revealed accumulation of deposits both in the Bowman layer and superficial stroma. On cross section, the deposits have a string of pearls appearance and some of them penetrate through the Bowman’s layer into the epithelium (Figure 3a). This can be seen clearer in an inverse modification of tomogram colouring with AS-OCT, considering the fact that the Bowman’s layer takes on a green color, as well as deposits in the classical tomograms (Figure 3b). The deeper layers of the stroma, Descemet’s membrane and the endothelium were normal (Figure 4a). Central corneal thickness was 531 μm on the right and 533 μm on the left measured by AS-OCT (Figure 4b).

Genomic DNA was extracted from EDTA-anticoagulated venous leucocytes using the PureLinkTM Genomic DNA Mini Kit (Invitrogen, Carlsbad, CA, USA). PCR products were purified by GeneJET PCR Purification Kit (Thermo Scientific) and then sequenced using the ABI Prism BigDye Terminator Kit (Applied Biosystems) on 3130 Genetic Analyzer (Applied Biosystems). The presence of genetic variations was analyzed using the Sequencing Analysis Software v5.2 Patch 2 (Applied Biosystems). Molecular genetic analysis of all exons of TGFBI gene showed a single heterozygous nucleotide substitution, G to A at the position 418 in the exon 4 and it did not identify other mutations (including R124C) in either of the cases. This mutation replaces amino acid arginine to histidine at codon 124 (R124H). The patient was not motivated for the surgical treatment of ACD, with pending consent for cataract surgery.

### Case 2

**Figure 5:**
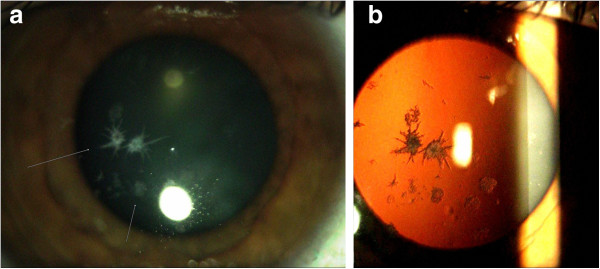
**Slit lamp photography in the left eye (Case 2).** The arrow indicates intense white snowflake-like deposits and rare gray-white granular deposits in mid-stromal layer **(Panel a)**. Retroillumination of corneal deposits **(Panel b)**.

**Figure 6:**
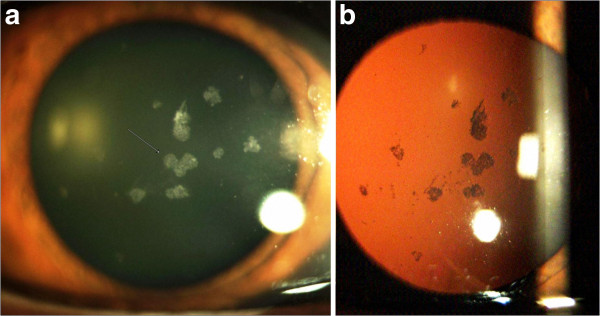
**The arrow indicates (Panel a) central heart-like deposits, while linear opacity absent.** Retroillumination of the right eye **(Panel b)** shows central placed deposits, with an absence of these deposits in the peripheral cornea.

**Figure 7:**
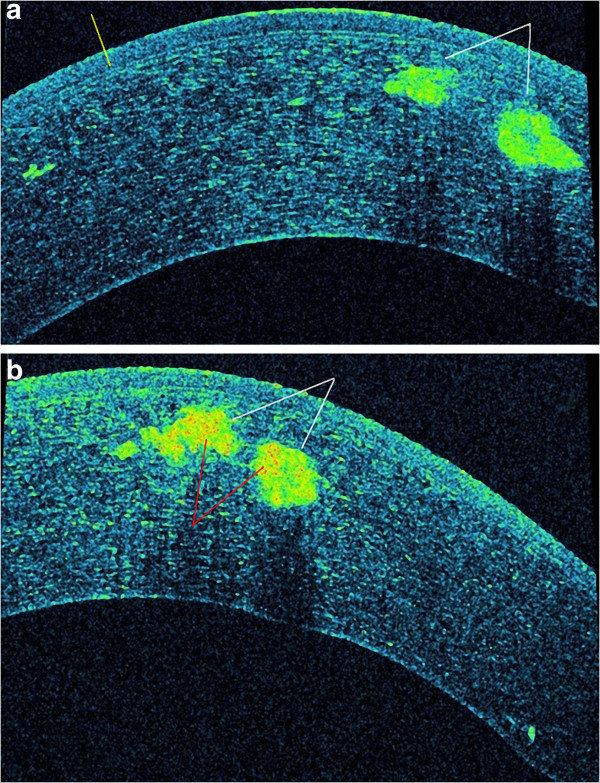
**AS-OCT asterisk scan (Case 2) shows large hyper-reflectivity deposits which are located at mid-stromal layer.** The epithelium, Bowman's layer, Descemet's membrane and endothelium appeared normal **(Panel a)**. The deposits at its central part with the hyper-reflection in relation to the periphery **(Panel b)**.

A 36-year-old man, the son of Case 1, was first examined at another clinic in the year of 1992 and he was diagnosed with granular corneal dystrophy type 1. He complained of periodical blurred vision in both eyes. The patient’s medical history showed simultaneous occurrence of corneal dystrophy and type 1 diabetes mellitus (DM 1) during the second decade of life. The patient was on intensive therapy with human insulin analogues, four times per day. Slit lamp examination showed intense white snowflake-like deposits and rare gray-white granular deposits, mainly distributed in the mid-stromal layer in the left eye (Figure 5a, 5b). The examination of the right eye revealed central, large, heart-like deposits and a few gray-white granular deposits in the anterior stroma (Figure 6a). The deposits are primarily located in the central cornea, with an absence of these deposits in the peripheral cornea (Figure 6b). Linear opacities were absent, while the epithelium, Bowman's layer, Descemet's membrane and endothelium appeared normal in both eyes. The patients best corrected visual acuity was 6/6 in both eyes and intraocular pressure by GAT was 14 mm Hg in both eyes. Dilated fundus examination bilaterally revealed widely scattered retinal hemorrhages (dot and blot), cotton wool spots and rare hard exudates in the macula (Mild Non-Proliferative Diabetic Retinopathy). AS-OCT showed snowflake-like, hyper-reflective deposits located at different depths within the corneal stroma (Figure 7a). The hyper-reflectivity differed in the center and the periphery of the deposits (Figure 7b). Central corneal thickness was 541 μm bilaterally and peripheral corneal thickness in the temporal area was 469 μm in the right eye. Genetic screening showed heterozygous for R124H mutation.

## Conclusion

ACD is typically an anterior stromal dystrophy, which has large intra and/or interfamilial phenotypic variation. Atypical and rare clinical presentation of Case 1 is characterized by deposits placed in the sub-epithelial and Bowman's corneal layers. The recurrent corneal erosions are a rare clinical feature of ACD. Spectrum of clinical changes in corneal dystrophy associated with TGFBI gene may show an accumulation of deposits in the epithelium, Bowman’s layer and stroma regardless of the type of point mutations. SL Edelstein et al. describe the clinical presentation similar to ACD with genotype of lattice corneal dystrophy (R124C) [4]. A clinical finding of lattice and/or granular deposits due to possible errors in clinical diagnosis requires genetic determination of mutation types. The degree of phenotypic expression is likely determined by the regulation and control mechanisms of TGFBI gene, which have not been fully clarified to this date. Due to the variable phenotypic expression of KE, Han et al. suggest slit lamp examinations at frequent intervals in heterozygotes for the R124H mutation [5]. By reviewing the available literature, we have not found the occurrence of systemic diseases associated with ACD. In our case, we presented comorbidity with RA and DM1 in the patients with ACD. Several papers have described mutations of the TGFBI gene, which may be related to RA, DM, atherosclerosis and other systemic diseases [6,7]. As a relatively novel and non-invasive imaging technique of the anterior segment, ultra high resolution AS-OCT helps us demonstrate different morphologic characteristics of ACD in vivo. To our knowledge, this is the first report describing ACD in Southeastern Europe, the Balkans and Serbia.

## Consent

Written informed consent was obtained from the patient for publication of this Case report and any accompanying images. A copy of the written consent is available for review by the Series Editor of this journal.

## Abbreviations

OMIM: Online Mendelian inheritance in man®; TGFBI: Transforming growth factor, beta-induced; AS-OCT: Anterior segment optical coherence tomography; GAT: Goldmann applanation tonometer; EDTA: Ethylenediaminetetraacetic acid; PCR: Polymerase chain reaction.

## Competing interests

The authors declare that they have no competing interests.

## Authors’ contributions

ZA Patient interaction and diagnosis, participated in interpreting the data, drafting of manuscript, final approval of manuscript. LM Patient interaction and diagnosis, final approval of manuscript. IG Patient interaction and diagnosis conducted a genetic analysis and edited the manuscript. DR Provided clinical information and participated in its design, final approval of manuscript. All authors read and approved the final manuscript.

## Pre-publication history

The pre-publication history for this paper can be accessed here:

http://www.biomedcentral.com/1471-2415/13/30/prepub
